# Prison healthcare service use and associated factors: a cross sectional study in Northwestern Ethiopia

**DOI:** 10.3389/fpsyt.2024.1426787

**Published:** 2024-08-06

**Authors:** Yassin Mohammed Yesuf, Amlaku Alemu Birhan, Addisu Gedlu Birara, Bewket Dereje Adimas, Abebe Bahiru Bezabh, Nega Gedefaw Agmase

**Affiliations:** ^1^ Department of Psychology, College of Social Sciences and Humanities, University of Gondar, Gondar, Ethiopia; ^2^ Department of Social Anthropology, College of Social Sciences and Humanities, University of Gondar, Gondar, Ethiopia; ^3^ Department of Criminology and Criminal Justice, College of Social Sciences and Humanities, University of Gondar, Gondar, Ethiopia; ^4^ Department of Law, School of Law, University of Gondar, Gondar, Ethiopia

**Keywords:** healthcare service utilizations, medical service use, guidance and counseling service use, psychiatric service use, prisons, inmates, Northwestern Ethiopia

## Abstract

**Introduction:**

Studies on inmates’ Health Care Service (HCS) utilization are scarce globally, infrequent in Ethiopia while findings about the factors associated with HCS utilization are inconsistent. The present study, therefore, examined inmates’ HCS utilization and associated socio-demographic and imprisonment related factors in Northwestern Ethiopia.

**Methods:**

The study employed institution-based cross sectional research design and data was collected using questionnaire from 422 inmates in three prisons. The questionnaire collected data about prisoners’ demographic characters, imprisonment related information and HCS utilization. Descriptive statistical techniques as well as bi-variate and multiple logistic regressions were used to analyse the data.

**Results and discussions:**

The study found that 72.5%, 66.1% and 13.3% of the inmates, respectively, used medical services, guidance and counseling services, and psychiatric services. Inmates with primary education, with secondary education, and who know the availability of the services were more likely to use medical services. Inmates with accused status were less likely to use medical services than inmates with convict status. Divorced marital status and knowledge of the service availability were associated with high guidance and counseling service use. An increase in the length of stay in the prison was associated with a decrease in psychiatry service use while knowledge of service availability was associated with higher odds of psychiatry service use. There are high medical care service utilization while low mental health care service utilization among inmates in Northwestern Ethiopia. Results of the study implied that there is a critical need for immediate health care service promotion and education measures. Besides, there are also needs for large scale, longitudinal and potentially cross-cultural studies to better understand additional factors that influence inmates’ HCS utilization.

## 1 Introduction

Globally, there are 11.5 million people in prison (1). The prison population is growing worldwide; in Africa this growth was 15% between 2000 and 2015 (2). By its very nature, incarceration can be deleterious to a prisoner’s mental and physical well-being. And the world health organization pinpointed that “Prisoners tend to have poorer physical, mental and social health than the population at large” (3, 15). In fact incarcerated individuals have elevated rates of infectious diseases and mental health issues (4). As a result the health of inmates has become a public health issue that need to be properly addressed.

Therefore, prison administrators have initiated a variety of rehabilitation programs that would enhance the overall wellbeing of inmates. Technically, the mere presence of the service doesn’t guarantee participation (5, 6). For example, in a Canadian research, 18.4% of the inmates who reported a recent history of mental illness or self-harm risk or distress did not get treatment (6). So, it is imperative to look into inmates’ actual participation in prison services.

Examining the participation of prisoners in Health Care Services (HCS) is important because participation has been linked to a lower recidivism rate. For instance, participation in rehabilitation treatment programs (7); psychological interventions (8); and psychiatric treatment at specialized forensic outpatient clinic (9) were associated with decreased recidivism rates.

Meanwhile, existing studies focused on the outcomes of participation (10), while studies on inmates’ participation rates in HCS and on why inmates do not participate are scarce (11–13). The available studies, mainly from the USA and Europe, reported different rates of participation. For instance in a study in Switzerland 82% the inmates utilized HCS from GPs, 90% of them utilized HCS from nurses and 43% of the inmates participate in psychiatric services (14). A study conducted in the USA found that 22% of the prisoners took part in mandated counseling sessions, while 31% of them participated in the voluntarily counseling service (15).

The HCS in prisons in Africa are poorly organized and are of poor quality. For example in a qualitative study in Ghana, there are no separate rooms designated for providing guidance and counseling services thereby difficult to maintain confidentiality. And professionals with insufficient experience offer guidance and counseling services (16). A scoping review of studies involving female prisoners in Sub-Saharan Africa (SSA) revealed that prison health facilities fall short of international standards. Plenty of barriers restrict inmates’ access to HCS outside of prisons (17). Similar review of studies among young inmates (aged 12 and 18) in SSA described the HCS as inadequate and alarmingly poor. Consequently, numerous obstacles impede prisoners’ access to healthcare both within and outside of prisons (18). This being the fact, there aren’t many studies on HCS utilization in African contexts. Such studies are particularly important in SSA where inmates “suffer more from inequitable access to healthcare services” (19, 101).

Meanwhile, findings about the factors associated with HCS utilization are inconsistent (12). Socio-demographic and imprisonment related factors were examined as factors associated with HCS utilization but the exact effects of the variables remain unclear and contradictory findings are reported across many research. A good example from the socio-demographic variables is gender. In a study in Switzerland (20) it was depicted that females were more likely to use mental health services than their male counterparts. In another study in Switzerland (14) females were less likely to use psychiatric services. Contrary to these findings, two studies conducted in Norway (21, 22) found no differences in HCS based on gender.

Likewise, length of stay is one of the imprisonment related factors associated with inmates’ HCS participation where contradictory findings are reported. In one of the studies in Switzerland (20) longer stay was associated with increased HCS use while in a longitudinal study in Portugal (12) it was associated with decreased HCS utilization. These all suggested that findings related to the factors associated with inmates’ participation in prison HCS are inconsistent thereby require additional investigations.

Furthermore, it was found that informational barriers were the main deterrents to participating in prison programs, particularly those related to education and vocational training (e.g. see 23). Likewise, in studies among university students in Ethiopia and elsewhere it was consistently found that lack of information about where to go is an important barrier to HCS use (e.g. (24–26). Meanwhile, giving information about the availability of HCS in prisons in African context is recommended (19). However, little research was done on these barriers in relation to prisoners’ use of HCS. It is, therefore, valuable to examine inmates’ knowledge of service availability or lack of knowledge thereof as a variable associated with inmates’ HCS utilizations.

In the Ethiopian context, the Ministry of Health (MoH) labeled prison inmates as “special vulnerable groups” that require special healthcare service (27). According to Article 28 of Federal Prison Proclamation No. 1174/2019, prison administrators are required to arrange HCS to prison inmates. The proclamation’s article 37 states that prisons medical services, including general health care, mental health care, and psychiatry services should be established and operated in prisons. As per the proclamation’s Article 42, prisons are required to offer psychological treatment to inmates as soon as they are accepted (28).

In actuality, many Ethiopian prisoners suffer from various physical and mental health conditions. In a meta-analysis and systematic review in Ethiopia 53.40% of inmates were found to have depressive symptoms (29). In another meta-analysis and systematic review, the prevalence of Tuberculosis (TB) among prison inmates in Ethiopia was found be high (30). Cognizant of this fact, a cross-sectional study depicted that 56.6% of inmates in Kality prion have depressive symptoms (31). Likewise, inmates in Northwestern Ethiopia were found to have higher rates of mental illnesses (32), suicidal ideation and attempt (33).

While these are the facts on the ground, studies on HCS in Ethiopian prison systems are infrequent. The few studies that are currently available looked at the delivery of HCS. For instance, a study conducted in Southern Ethiopia on the general rehabilitation program revealed that there are comparatively few prison treatment staff members compared to a large number of security personnel (34). In the Oromia National Regional State of Ethiopia, a research was carried out to look at the rights of inmates in the Ilu Abba Bor and Buno Bedelle Zones. According to the study, some prison centers had no medical services, while those who did (clinics per see) faced a variety of difficulties. It was also indicated that the clinics have lack budget, equipment, and transport services to nearby hospitals (35). These studies only examined the HCS provision and depicted that the HCS in prisons are inadequate.

The inadequate healthcare services in Ethiopian prisons alongside the high rate of mental and physical illness among prisoners deserve due attention. It is particularly important to understand how many prisoners are using the HCS and, if not, why they are not using the services. These types of studies are difficult to find in Ethiopia. The only exception here is a qualitative study in Southern Ethiopia that examined the standard healthcare utilization of inmates with HIV/AIDS. The study indicated that inmates HCS utilization is limited because of structural and social barriers such as insufficient support from HCS staff, uncooperative security system, lack of patient privacy, social stigma that prevents inmates from disclosing their status, and lack of food supplies (36). However, this study is delimited service utilization related to HIV/AIDS. The present study, therefore, attempted to fill the aforementioned research gaps by focusing on the HCS (Medical Service, Guidance and Counseling Service, and Psychiatric Service) utilization of inmates in Northwestern Ethiopia.

Based on research results that revealed poor HCS utilization in the general population (e.g. 37, 38), we first hypothesized that there will be low HCS utilization among prisoners. It was also hypothesized that sociodemographic and imprisonment-related factors, including inmates’ knowledge of service availability, will influence inmates’ participation in HCSs.

The results of this study will help policy makers, prison administrators, and other stakeholders to develop customized interventions that may increase prisoners’ use of the available HCS. This would ultimately reduce recidivism in the nation by enabling prisoners to reap the advantages of improved health that come with participating in the programs. Additionally, our study’s findings will contribute to the global body of knowledge on inmates’ HCS use and associated socio-demographic and imprisonment related factors from a non-Western prison setting.

## 2 Materials and methods

### 2.1 Research design

The present study employed institution-based cross sectional research design where quantitative data collection technique is used. In terms of its methods of analysis, the study employed both descriptive and explanatory research designs. It is descriptive in that it summarized and described respondents’ characteristics, HCS utilizations and imprisonment related factors including their knowledge of the available services. It is explanatory because it tests the associations that existed between inmates’ HCS utilization and predictor variables.

### 2.2 Setting

Amhara National Regional State is home to 31 correctional facilities, 30 of which fall under regional administrative control and the remaining one facility (Shewarobit Rehabilitation and Correction Center) is under federal administrative control. There are two levels of correctional facilities in the region: higher level (12 in total), and medium and lower level (18 in total).

Of the 30 prisons in the region, 10 of them are found in the North-western part of the regional state. Simple random sampling technique was used to select three prisons: Gondar, Debretabor and Bahirdar prisons. While Debretabor Correction Center is under lower and medium level centers, Gondar and Bahirdar Prisons are higher level centers. During data collection there were 2417 inmates in Gondar prison, 2648 inmates in Bahirdar prison and 2099 inmates in Debretabor prison.

### 2.3 Healthcare services in the prisons

The Federal Prison Proclamation enforces prison administrations to provide health care services to inmates (28). Hence, there are clinics in the three prisons run by nurses and provide primary health care services. For complicated medical cases, the clinics have referral attachments with the nearby government hospitals in their respective cities. Besides, there are offices for guidance and counseling services inside the three prisons run by psychologists. The medical and counseling service provisions are initiated by the inmates and thus are voluntary. There are no psychiatric services per se in the three prions. Inmates receive psychiatric services in the nearby hospitals through referrals from the psychologists.

### 2.4 Sample size

For the purpose of determining the sample size of the study single proportion formula was used (n = [Z²a/2 X p(q)]/d²) with 95% confidence interval, a proportion of 50% (0.5) and e= + 0.05. Based on the computations using the formula the minimum sample size was 384. Assuming 10% non-response rate the final sample size was 422. During data collection there were 2,648 inmates at Bahirdar prison, 2,417 inmates at Gondar prison and 2,099 inmates at Debretabor prison. Quota sampling was used to include proportional number of inmates from the three prisons. Therefore, 142, 124 and 156 inmates from Gondar, Debretabor and Bahirdar prisons, respectively, participated as questionnaire respondents. Simple random sampling was used to select participants from each correction center.

### 2.5 Measures

In the present study data was collected using a structured and pretested questionnaire. The questionnaire has three sections. The first section collects data about inmates’ demographic characteristics while the second section collects imprisonment related data. Inmates’ HCS utilization measures are included in the third section of the questionnaire.

Inmates’ demographic factors: based on findings from earlier studies, age (measured in years); gender (male or female), educational status (the highest educational level attended); marital status (married, single/never married or divorced); and inmates’ employment status before they were incarcerated (categorized into unemployed, employed by others or self-employed) were included as demographic factors.

Imprisonment related factors: the length of stay (measured in years); frequency of imprisonment (first time or recidivist); convict status (pretrial detainees, accused or Sentenced); and type of offense the prisoner is serving (categorized as crime against person, crime against property and crime against state) were imprisonment related factors included in the present study. In addition, it is believed that an individual’s behavior (participation in our case) is dependent on his/her knowledge. We have included inmates’ knowledge of the three HCS as imprisonment related factor. Inmates were therefore asked to report whether they know the availability of the three HCS offered at the facility they are incarcerated. These were all dichotomous indicators of whether they had knowledge (1 = know, 0 = don’t know) of the three services offered at the facility.

Service Utilization: Inmates were asked to report whether they participated in the medical, guidance and counseling and psychiatry services offered in their respective facilities or not. These were all dichotomous indicators of whether they participated (1 = yes) or not (0 = no) in each types of services offered at their respective facilities.

### 2.6 Data collection procedures and ethical considerations

The questionnaire was prepared in English and translated to Amharic by experts in law, psychology, criminology and language. It was then back translated by public health, criminology and language experts who were not familiar with the purpose of the study. And minor differences in translations were resolved through a focus-group discussion. Formal letters directed to the selected facilities were written from the college of social science and humanities requesting permission to collect data at the respective facility. While delivering the letters, the purpose of the research was vividly communicated to prison administrators. At individual participant level, the participants were informed about the purpose of the study and measures were taken to maintain the respect, dignity and freedom of each individual participant and every inmate was assured about confidentiality. Participants were also ensured that they have full right to discontinue or refuse to participate in the study.

This study is part of a mega-project that examined the situation of prisons in Northwestern Ethiopia (The Prison Project). A representative from the regional prison administration office was with us while we develop the proposal. After the three prisons are selected, we have also included one prison staff from each prison as a focal person. The prison staffs in each prison helped the data collectors in selecting inmates. Data collection process was carried out by six trained M.A. holders (who were research team members and have prior experience in mixed-methods research data collection). Two data collectors on each facility contacted the inmates personally and assist them to complete the questionnaire. The data collectors handed the questionnaire to the inmates and the questionnaire was read for those who can’t read and write. And data collection was conducted either individually or in group meetings. Each questionnaire was immediately checked for completeness and if the questionnaire turned out to be incomplete, it was substituted with another questionnaire data from a different prisoner. Issues related with how crime types committed are categorized; the inclusion and exclusion criteria; and other data collection procedures are included elsewhere (32).

### 2.7 Methods of data analysis

Both descriptive and inferential statistics were used in the present study. Frequencies and percentages were computed to describe respondents’ demographic characteristics, imprisonment related factors, inmates’ knowledge of HCS available and HCS utilization among inmates. Moreover, mean standard deviation, maximum and minimum scores were computed to describe continuous variables (age and length of stay). Prison Healthcare service participation is dichotomous (participate or not participate) thereby logistic regression is performed. Preliminary tests were conducted to check for multicollinearity, outliers, and goodness of fit assumptions. Results showed that there were no major violations of these assumptions. So, the influence of demographic characteristics and imprisonment related factors on healthcare utilization was explored initially using bivariate analyses. Significant covariates of healthcare use were then added to the conditional models (multiple logistic regression models). A 95% confidence interval was used for the parameter estimates. All data analyses were carried out using SPSS version 23.

## 3 Results

### 3.1 Description of the respondents

As can be seen from **Table 1** respondents are aged between 16 and 67 years with a mean age of 31.91 years (SD= 11.083). The respondents stayed between 0 and 13 years in the prison where the mean stay of the respondents is 1.51 year (SD= 1.751). Majority of the respondents are males (92.2%), attend secondary education (39.3%), married (48.6%) and employed (42.7%). In terms of imprisonment related characteristics, majority of the respondents are imprisoned once (95.7%), have sentenced status (85.5%), and committed crimes against person (57.8%).

**Table 1:** Demographic and imprisonment related characteristics of the respondents (N=422).

Variables	Category	Mean (SD)	Min(Max)	N	%
Age		31.91 (11.083)	16 (67)		
Length of Stay		1.52 (1.751)	0 (13)		
Gender	Male			389	92.2
	Female			33	7.8
Educational Status	No Schooling			81	19.2
	Primary Education (Grades 1-8)			147	34.8
	Secondary Education (Grades 9-12)			166	39.3
	Higher Education			28	6.6
Marital Status	Single			195	46.2
	Married			205	48.6
	Divorced			22	5.2
Employment Status	Unemployed			63	14.9
	Employed			180	42.7
	Self-employed			179	42.4
Frequency of imprisonment	First time			404	95.7
	Recidivist			18	4.3
Convict status	Pre trail			23	5.5
	Accused			38	9.0
	Sentenced			361	85.5
Types of Crime	Crime Against Person			244	57.8
	Crime against Property			123	29.1
	Crime Against State			55	13.0

### 3.2 Inmates’ knowledge and utilization of healthcare services

**Table 2** depicted that 85.1% of the inmates know the presence of medical services while 72.5% of them use prison medical services. Likewise, 87.4% of the inmates know the availability of guidance and counseling services and 66.1% of the inmates use the services. With regard to psychiatry services, 29.4% of the inmates know the presence of the service while 13.3% of them utilize psychiatry services.

**Table 2:** Respondents’ knowledge and use of health care services.

Available Service	Know		Don’t know		Use		Don’t use	
	N	%	N	%	N	%	N	%
Medical services	359	85.1	63	14.9	306	72.5	116	27.5
Guidance and Counseling services	369	87.4	53	12.6	279	66.1	143	33.9
Psychiatric services	124	29.4	298	70.6	56	13.3	366	86.7

### 3.3 Factors associated with inmates’ health care service utilization

#### 3.3.1 Factors associated with medical service use

In the bivariate analysis education, convict status, types of crime committed and knowledge of medical service availability were significantly associated with medical service use (see **Supplementary Material 1** for all variables). In the multiple logistic regression analysis all, except types of crimes committed, were significantly associated with medical service use. Specifically, **Table 3** indicated that inmates with primary education (AOR=2.256, p<0.05, 95% CI= 1.155, 4.408) and inmates with secondary education (AOR= 1.955, p<0.05, 95% CI=1.012, 3.779) were two times more likely to use the medical services than inmates who didn’t get schooling. With regard to inmates convict status, inmates with accused status (AOR=.386, p<0.05, 95% CI= .183,.811) were less likely to use medical services than inmates with convict status. Inmates who know the availability of medical services (AOR=8.103, p<0.01, 95% CI= 4.405, 14.903) were 8 times more likely to use the services than inmates who don’t know the availability of the services.

**Table 3:** Factors associated with Medical service use.

Variable	Category	OR	p	AOR (CI)	p
Educational status	No schooling	1		1	
	Primary education	2.170	.010	2.256 (1.155-4.408)	.017
	Secondary education	1.991	.018	1.955 (1.012-3.779)	.046
	Higher Education	1.379	.489	1.503 (.570-3.962)	.410
Convict status	Sentenced	1		1	
	Accused	.327	.001	.386 (.183-.811)	.012
	Pre trail	.614	.282	1.253 (.437-3.592)	.675
Types of crime	Against Person	1		1	
	Against Property	2.242	.004	1.692 (.907-3.154)	.098
	Against State	.653	.167	.767 (.391-1.502)	.439
Knowledge about service availability	Don’t know	1		1	
	Know	7.057	.000	8.103 (4.405-14.903)	.000

#### 3.3.2 Factors associated with guidance and counseling service use

As can be seen from **Table 4**, education, marital status and knowledge of the availability of guidance and counseling services were significantly associated with service use in the bivariate analysis (detailed results can be found in **Supplementary Material 2**). In the multiple logistic regressions analysis, marital status and knowledge of the availability of guidance and counseling services remain significant. The odds of using the guidance and counseling services among inmates who are divorced were seven times higher than the odds of using the services among single inmates (AOR= 7.015, p<0.05, 95% CI=1.524, 32.290). Inmates who know the availability of guidance and counseling services were 4 times higher in using the services than inmates who don’t know the availability of the services (AOR= 4.169, p<0.01, 95% CI=2.224, 7.817).

**Table 4:** Factors associated with Guidance and Counseling service use.

Variable	Category	OR	p	AOR (CI)	p
Educational Level	No schooling	1			
	Primary education	.954	.877	.908 (.484-1.705)	.764
	Secondary education	.764	.356	.879 (.468-1.654)	.690
	Higher Education	.365	.025	.386 (.148-1.010)	.052
Marital status	Single	1		1	
	Married	1.206	.372	1.389 (.878-2.204)	.163
	Divorced	5.984	.018	7.015 (1.524-32.290)	.012
Knowledge about service availability	Don’t know	1		1	
	Know	4.268	.000	4.169 (2.224-7.817)	.000

#### 3.3.3 Factors associated with psychiatry service use

**Table 5** presents factors associated with psychiatry service use. In the bivariate analysis, length of stay in the prison and knowledge of psychiatry service availability were significantly associated with psychiatry service use (results about the remaining factors are included in **Supplementary Material 3**). These factors remain significant in the multiple logistic regression analyses. An increase in the length of stay in the prison is associated with a decrease in psychiatry service use (AOR= .455, p<0.01, 95% CI=.311,.665). Besides, inmates who know the availability of psychiatry services were found to have higher odds of psychiatry service use than inmates who didn’t know the availability of the service (AOR= 29.028, p<0.01, 95% CI= 12.363, 68.159).

**Table 5:** Factors associated with psychiatry service use.

Variable	Category	OR	p	AOR	p
Length of stay		.451	.000	.455 (.311-.665)	.000
Knowledge about service availability	Don’t know	1		1	
	Know	27.160	.000	29.028 (12.363-68.159)	.000

## 4 Discussions

This study examined inmates’ HCS utilizations and associated factors in Northwestern Ethiopia. The study is the first of its kind to examine inmates’ HCS utilization in Ethiopia, at least to the knowledge of the present researchers. The study’s findings will also be important because it illustrates the significant impact that inmates’ knowledge of service accessibility plays in their use of HCS. This is because it is anticipated that the findings will spark scholarly discussion among experts in HCS use research.

In this study we first aimed to assess the knowledge inmates have about health care services available. While they are less aware of psychiatric services’ availability, inmates are well-informed about the availability of medical care services as well as guidance and counseling services. The low knowledge of psychiatric services among inmates could be attributed to the fact that the services are given in nearby hospitals. Likewise, the high knowledge of inmates on the medical care services and guidance and counseling services could be attributed to the facts that the services are given inside the prisons. The prison proclamation advocate for the provision of healthcare services as well as the delivery of health education activities by prison administrators (28). The fact that there are inmates who didn’t know the availability of the healthcare services reflects the problems in the health education activities in the prisons in Northwestern Ethiopia.

These findings implied that it is improper to believe that inmates will know the presence of the services as long as the services are available. Similar findings are reported in a study in Nigeria. In the study all the inmates reported that they have knowledge of rehabilitation services available in the prison but none of them stated health care services (39).

Second, we examined inmates’ participation levels in the three health care services and similar trends to their knowledge of the services are found. The medical health care utilization is said to be high. The medical service utilization in our sample is high compared to the reported 41.8% of health care utilization in a community based study in Dessie city, Amhara region (38). Likewise, a study in East Gojam, Amhara region, reported a rate of 41.5% HCS utilization (37) while another study in Achefer, Northwest Ethiopia, reported a rate of 39.9% (40) which implies that the rates of medical care utilization in our study is high. On the other hand, higher medical care utilization comparable to our study is reported among inmates in Switzerland (14) where 82% the inmates utilized health care services from GPs and 90% of them utilized health care services from nurses. The high medical care utilization rate in our study could be attributed to high rates of illnesses reported from other studies, e.g. high rates of TB among inmates (30).

With regard to guidance and counseling service use, it is found that 61.6% of the inmates utilize the service. Given the high number of inmates who are knowledgeable about the service availability (87.4%) the service utilization cannot be regarded as high. On the other hand, the guidance and counseling service utilization could also be considered as high when compared with the service utilization in other settings. For example, in a country where only 2% of university students in Amhara region are using guidance and counseling services (41) the participation rate found in our study is high. Coupled with this, the utilization rate could also be regarded as high when compared with findings from other studies. For instance, in a study in the USA it was revealed that 31% of the inmates voluntarily participated in the counseling service while 22% of them participated in mandated counseling services (15).

The psychiatry service utilization is low but it was expected. This is because the services are given in the nearby hospitals to individuals with severe illnesses; psychiatric services in Ethiopia are characterized low doctor-patient ratio, one psychiatrist serving 6 million individuals (42); and many Ethiopians prefer informal help for mental illness treatment (43).

Higher rates of psychiatric service utilization were reported from other studies. The study in Switzerland reported that 43% of the inmates participate in psychiatric services (14), a rate which is higher than the rate found in our study. Another study in Norway examined inmates’ participation in mental health consultation and reported a rate of 25%, which is still higher than what is found in our study. However, the study also found that it is only 3% of the inmates who received treatment for psychotic disorders (21). The difference could be attributed to the fact that psychiatric service to inmates in Ethiopia are delivered in the nearly hospitals while in both Switzerland and Norway the services are given inside the prisons. Coupled with this, the variations on what constitute psychiatric services could also be a factor for differences in findings. In our study we have assessed the psychiatric services inmates received in the nearby hospitals through referrals from counselors in the prisons. The study in Switzerland assessed psychiatric services rendered inside the prison only (14). On the other hand, the study in Norway assessed the services inmates received from psychologists, psychiatric nurses and psychiatrists in the prisons as well as the emergency psychiatric services the inmates received from private psychologists/psychiatrists and a deviant sexuality treatment clinic (21).

When we critically look into the overall mental health care utilizations (the guidance and counseling service utilization and psychiatry service utilization combined), there are low service utilization in the present study. This finding is similar with a research finding that depicted that there are low mental health care service utilizations in Ethiopia because of stigma and discrimination associated with mental illnesses (44).

In the present study factors associated with health care service utilization were also examined. Knowledge of service availability was consistently found to be associated with inmates’ health care service utilization. Inmates who have the knowledge of medical care services, guidance and counseling services and psychiatric services utilize the services more than the inmates who didn’t have the knowledge. In the global literature low health literacy is associated with high HCS utilization (45, 46). Given the strong association between health literacy and health knowledge (47), one could argue that health knowledge and HCS utilization will have negative associations. Our findings are against this argument indicating that knowledge of service availability and utilization have positive associations. This implied that the role of knowledge of service availability on HCS utilization still needs further investigations. This is because knowledge of service availability may not entail general health knowledge.

Of the other variables included in the present study, education, marital status, convict status and length of stay were associated with the different health care service utilizations.

In the present study inmates who are sentenced were more likely to use medical services than inmates who were in accused status. Contrary to this finding, in the two studies in Switzerland inmates with pretrial detentions were found to use more nursing and GP health care services (14, 20). However, the study in Norway that examined inmates’ participation in medical and mental health services found a similar result with our study where sentenced inmates were found to use more services than inmates in pretrial detention (22). The possible explanation for our finding is that inmates who are only accused might think of their release soon thereby delay their medical care use in the prison. Given the poor quality medical care in the prisons in Ethiopia (35) inmates who are accused might delay their service use while the only option for sentencedinmates is using the prison medical services.

Inmates’ educational status was associated with medical service use. Specifically inmates who attended primary and secondary education were more likely to participate in medical services than inmates with no schooling. Contrary to this finding, in the study in Norway education was not found to be associated with inmates’ participation in medical and mental health services (22). The small number of inmates with no schooling and with higher education status in our study could explain the variation in the findings. In the community based study in Achefer inmates with primary and above primary schooling were more likely to use medical health care service than inmates who can’t write and read (40). This finding substantiates the results from our study that depicted that inmates’ educational status is one of the significant factors in their medical care utilizations.

Inmates who are divorced were found to utilize more guidance and counseling services than inmates who are single. The study in Switzerland categorized inmates as married and unmarried where the former use less psychiatric services than the later (14). Differences in findings could be attributed to how the studies categorized inmates’ marital status. The negative effects of the divorce itself might have induced psychological problems and that in turn might have increased the odds of inmates in the present study to use the guidance and counseling services.

Length of stay is associated with psychiatric service utilizations where longer stay is associated with lower odds of psychiatric service utilizations. Similar to this finding, the study in Switzerland found an association between longer stay in prison with lower psychiatric service utilizations (14). From this findings it can be deduced that as inmates stay longer in prison they might adapt to the distresses because of the incarcerations thereby lower odds of using the psychiatric services. The fact that people with psychiatric disorder are difficult to find could also be a possible explanation here.

The cross-sectional nature of the study alongside with the fact that the study includes three prisons only could be considered as the limitations of the study. Moreover, the researchers acknowledged that the study would have been better had it included additional variables like having children, place of residence, presence of illnesses, etc. We also acknowledged that other important variables that could potentially influence prison HCS utilization are missing. For instance location of the service, such as whether it is offered inside or outside of a prison, may have an impact on the use of HCS. Similarly variables like treatment needs were not investigated. Future research need to include service location, treatment need and other important variables.

## 5 Conclusions and recommendations

There are high medical healthcare utilizations while there are low levels of mental health care service utilizations in the prisons in Northwestern Ethiopia. Significant numbers of inmates are unaware of the available HCS in their prisons. All of these suggested that there is much work to be done to enhance the way that prisoners use HCS, and consequently, their health. Mainly, health care service promotion and education measures need to be conducted by involving stakeholders including inmates, prison personnel, community representatives, health care professionals, police makers, etc. This is because knowledge of HCS availability was found to be the key factor influencing inmates’ usage of the HCS.

The factors associated with inmates’ use of medical services and the factors associated with their use of mental health services are different. Therefore, it is necessary to implement different approaches accordingly.

Above all, the results of this study can be used by policy makers, prison administrators, and other relevant organizations to create customized interventions that will increase inmates’ use of HCS, better their mental and physical health, and ultimately reduce recidivism in Ethiopia. Given the scope of the study is delimited to Northwestern Ethiopia; it is recommended that nationwide study, possibly a longitudinal one, need to be conducted. In the present study relatively high medical care utilization by inmates than the utilization rates reported from studies in the general public is found. Comparing the HCS utilization of inmates with the HCS utilizations by general public is, therefore, an important future research area in Ethiopia.

The current study assessed general participation in HCSs using a quantitative approach. Thus, extensive details regarding the involvement of prisoners are not investigated. It is possible to assess HCS use details using qualitative approaches. Future research therefore requires a mixed method research in order to mitigate the natural shortcomings of qualitative as well as quantitative methods.

## Data availability statement

The original contributions presented in the study are included in the article/**Supplementary Material**. Further inquiries can be directed to the corresponding author.

## Ethics statement

The studies involving humans were approved by College of Social Science and Humanities Research Review Committee. The studies were conducted in accordance with the local legislation and institutional requirements. The participants provided their written informed consent to participate in this study.

## Author contributions

YY: Conceptualization, Formal analysis, Methodology, Project administration, Supervision, Validation, Writing – original draft, Writing – review & editing. AB: Conceptualization, Formal analysis, Funding acquisition, Methodology, Project administration, Writing – original draft, Writing – review & editing. AB: Conceptualization, Formal analysis, Methodology, Project administration, Validation, Writing – original draft, Writing – review & editing. BA: Conceptualization, Formal analysis, Investigation, Methodology, Project administration, Validation, Writing – original draft, Writing – review & editing. AB: Conceptualization, Methodology, Project administration, Validation, Writing – original draft, Writing – review & editing. NA: Investigation, Methodology, Validation, Writing – original draft, Writing – review & editing.
