# Supplementary material for: Prison healthcare service use and associated factors: a cross sectional study in Northwestern Ethiopia
Source: Front Psychiatry. 2024 Aug 6;15:1426787. doi: 10.3389/fpsyt.2024.1426787 (PMC11337193; doi:10.3389/fpsyt.2024.1426787)
Supplement: Supplementary file 1 [file Table_1.doc]

**Supplementary Material 1**

**Bivariate and multiple logistic regression of demographic and imprisonment related factors on Medical Service utilization**

| **Variable** | **Category** | **OR** | **p** | **AOR** | **p** | **95% CI** | |
| --- | --- | --- | --- | --- | --- | --- | --- |
|  |  |  |  |  |  | **Lower** | **Upper** |
| Sex | Male | 1.352 | .435 |  |  |  |  |
|  | Female | 1 |  |  |  |  |  |
| Age |  | 1.001 | .880 |  |  |  |  |
| Educational Level | No Schooling | 1 |  | 1 |  |  |  |
|  | Primary Education | 2.170 | .010 | 2.256 | .017 | 1.155 | 4.408 |
|  | Secondary Education | 1.991 | .018 | 1.955 | .046 | 1.012 | 3.779 |
|  | Higher Education | 1.379 | .489 | 1.503 | .410 | .570 | 3.962 |
| Marital Status | Single | 1 |  |  |  |  |  |
|  | Married | .716 | .139 |  |  |  |  |
|  | Divorced | .847 | .743 |  |  |  |  |
| Employment Status | Unemployed | 1 |  |  |  |  |  |
|  | Employed | .764 | .437 |  |  |  |  |
|  | Self-employed | .679 | .261 |  |  |  |  |
| Length of Stay |  | .959 | .491 |  |  |  |  |
| Frequency of Imprisonment | First time | 1 |  |  |  |  |  |
|  | Recidivist | 1.336 | .571 |  |  |  |  |
| Convict status | Convicted | 1 |  | 1 |  |  |  |
|  | Accused | .327 | .001 | .386 | .012 | .183 | .811 |
|  | Pre trail | .614 | .282 | 1.253 | .675 | .437 | 3.592 |
| Types of crime | Against Person | 1 |  | 1 |  |  |  |
|  | Against Property | 2.242 | .004 | 1.692 | .098 | .907 | 3.154 |
|  | Against State | .653 | .167 | .767 | .439 | .391 | 1.502 |
| Knowledge about service availability | Don't know | 1 |  | 1 |  |  |  |
|  | Know | 7.057 | .000 | 8.103 | .000 | 4.405 | 14.903 |
