# Supplementary material for: Prison healthcare service use and associated factors: a cross sectional study in Northwestern Ethiopia
Source: Front Psychiatry. 2024 Aug 6;15:1426787. doi: 10.3389/fpsyt.2024.1426787 (PMC11337193; doi:10.3389/fpsyt.2024.1426787)
Supplement: Supplementary file 2 [file Table_2.doc]

**Supplementary material 2**

**Bivariate and multiple logistic regression of demographic and imprisonment related factors on Guidance and Counseling Service use**

| **Variable** | **Category** | **OR** | **p** | **AOR** | **p** | **95% CI** | |
| --- | --- | --- | --- | --- | --- | --- | --- |
|  |  |  |  |  |  | **Lower** | **Upper** |
| Sex | Male | 1.699 | .147 |  |  |  |  |
|  | Female | 1 |  |  |  |  |  |
| Age |  | 1.006 | .499 |  |  |  |  |
| Educational Level | No Schooling | 1 |  | 1 |  |  |  |
|  | Primary | 2.740 | .025 | .908 | .764 | .484 | 1.705 |
|  | Secondary | 2.615 | .022 | .879 | .690 | .468 | 1.654 |
|  | Higher education | 2.093 | .073 | .386 | .052 | .148 | 1.010 |
| Marital Status | Single | 1 |  | 1 |  |  |  |
|  | Married | 1.206 | .372 | 1.389 | .163 | .876 | 2.204 |
|  | Divorced | 5.984 | .018 | 7.015 | .012 | 1.524 | 32.290 |
| Employment Status | Unemployed | 1 |  |  |  |  |  |
|  | Employed | 1.154 | .655 |  |  |  |  |
|  | Self-employed | .599 | .102 |  |  |  |  |
| Length of Stay |  | 1.116 | .093 |  |  |  |  |
| Frequency of Imprisonment | First time | 1 |  |  |  |  |  |
|  | Recidivist | 2.015 | .147 |  |  |  |  |
| Convict status | Pre trail | 1 |  |  |  |  |  |
|  | Accused | .818 | .714 |  |  |  |  |
|  | Convicted | 1.071 | .879 |  |  |  |  |
| Types of crime | Against Person | 1 |  |  |  |  |  |
|  | Against Property | 1.218 | .406 |  |  |  |  |
|  | Against State | 1.031 | .922 |  |  |  |  |
| Knowledge about service availability | Don't know | 1 |  | 1 |  |  |  |
|  | Know | 4.268 | .000 | 4.169 | .000 | 2.224 | 7.817 |
