# Supplementary material for: Prison healthcare service use and associated factors: a cross sectional study in Northwestern Ethiopia
Source: Front Psychiatry. 2024 Aug 6;15:1426787. doi: 10.3389/fpsyt.2024.1426787 (PMC11337193; doi:10.3389/fpsyt.2024.1426787)
Supplement: Supplementary file 3 [file Table_3.doc]

**Supplementary material 3**

**Bivariate and multiple logistic regression of demographic and imprisonment related factors on Psychiatry Service Use**

| **Variable** | **Category** | **OR** | **p** | **AOR** | **p** | **95% CI** | |
| --- | --- | --- | --- | --- | --- | --- | --- |
|  |  |  |  |  |  | **Lower** | **Upper** |
| Sex | Male | 5.269 | .105 |  |  |  |  |
|  | Female | 1 |  |  |  |  |  |
| Age |  | 1.007 | .576 |  |  |  |  |
| Educational Level | No Schooling | 1 |  |  |  |  |  |
|  | Primary | .991 | .982 |  |  |  |  |
|  | Secondary | 1.319 | .489 |  |  |  |  |
|  | Higher education | .546 | .454 |  |  |  |  |
| Marital Status | Single | 1 |  |  |  |  |  |
|  | Married | 1.174 | .588 |  |  |  |  |
|  | Divorced | 1.125 | .858 |  |  |  |  |
| Employment Status | Unemployed | 1 |  |  |  |  |  |
|  | Employed | 1.187 | .726 |  |  |  |  |
|  | Self-employed | 1.913 | .171 | 1 |  |  |  |
| Length of Stay |  | .451 | .000 | .455 | .000 | .311 | .665 |
| Frequency of Imprisonment | First time | 1 |  |  |  |  |  |
|  | Recidivist | 2.679 | .343 |  |  |  |  |
| Convict status | Pre trail | 1 |  |  |  |  |  |
|  | Accused | .000 | .998 |  |  |  |  |
|  | Convicted | .456 | .115 |  |  |  |  |
| Types of crime | Against Person | 1 |  |  |  |  |  |
|  | Against Property | 1.102 | .765 |  |  |  |  |
|  | Against State | 1.170 | .714 |  |  |  |  |
| Knowledge about service availability | Don't know | 1 |  | 1 |  |  |  |
|  | Know | 27.160 | .000 | 29.028 | .000 | 12.363 | 68.159 |
